# Mathematical Model of Plasmid-Mediated Resistance to Ceftiofur in Commensal Enteric *Escherichia coli* of Cattle

**DOI:** 10.1371/journal.pone.0036738

**Published:** 2012-05-16

**Authors:** Victoriya V. Volkova, Cristina Lanzas, Zhao Lu, Yrjö Tapio Gröhn

**Affiliations:** 1 Department of Population Medicine and Diagnostic Sciences, College of Veterinary Medicine, Cornell University, Ithaca, New York, United States of America; 2 Department of Biomedical and Diagnostic Sciences, College of Veterinary Medicine, The University of Tennessee, Knoxville, Tennessee, United States of America; Massey University, New Zealand

## Abstract

Antimicrobial use in food animals may contribute to antimicrobial resistance in bacteria of animals and humans. Commensal bacteria of animal intestine may serve as a reservoir of resistance-genes. To understand the dynamics of plasmid-mediated resistance to cephalosporin ceftiofur in enteric commensals of cattle, we developed a deterministic mathematical model of the dynamics of ceftiofur-sensitive and resistant commensal enteric *Escherichia coli* (*E. coli*) in the absence of and during parenteral therapy with ceftiofur. The most common treatment scenarios including those using a sustained-release drug formulation were simulated; the model outputs were in agreement with the available experimental data. The model indicated that a low but stable fraction of resistant enteric *E. coli* could persist in the absence of immediate ceftiofur pressure, being sustained by horizontal and vertical transfers of plasmids carrying resistance-genes, and ingestion of resistant *E. coli*. During parenteral therapy with ceftiofur, resistant enteric *E. coli* expanded in absolute number and relative frequency. This expansion was most influenced by parameters of antimicrobial action of ceftiofur against *E. coli*. After treatment (>5 weeks from start of therapy) the fraction of ceftiofur-resistant cells among enteric *E. coli*, similar to that in the absence of treatment, was most influenced by the parameters of ecology of enteric *E. coli*, such as the frequency of transfer of plasmids carrying resistance-genes, the rate of replacement of enteric *E. coli* by ingested *E. coli*, and the frequency of ceftiofur resistance in the latter.

## Introduction

The emergence and spread of antimicrobial resistance (AMR) is progressively demarcating the epochal success of antimicrobial therapies of bacterial infections. Some classes of antimicrobials are used in both human and veterinary medicines; among antibiotics these are *β*-lactams, including cephalosporins, as well as aminoglycosides, macrolides, tetracyclines, sulphonamides, and in some countries fluoroquinolones [1]. Humans may be exposed to AMR-bacteria from food animals via occupational exposure or contaminated food products. In the 1990s in the USA, a domestically-acquired infection of a boy with ceftriaxone-resistant *Salmonella* was traced to cattle carrying ceftiofur-resistant *Salmonella* after the boy’s father had treated the diarrheic calves [2]. Human food-borne infections with AMR-bacteria are clinically challenging [1], [3]. Furthermore, ingested strains can become a part of the human enteric microflora [4], and transmit AMR-genetic determinants to other human bacteria [5]. For cephalosporins, the principal mechanism via which resistance disseminates is horizontal transfer of AMR-genes encoded on conjugative plasmids [6], [7], [8]. The AMR-strains occasionally demonstrate a higher transmissibility via the food chain, *e.g.*, an AMR-strain of *Escherichia coli* (*E. coli*) on pig carcasses has survived processing and chilling better than the parental antimicrobial-sensitive strain [9]. Cattle meat products can be contaminated by animals’ feces, and so the enteric microflora [10]. Therefore, minimizing the frequency of AMR in cattle enteric bacteria *en masse* can aid in decreasing human exposure to AMR-strains.

Within animal hosts, enteric commensals may also transmit AMR-genetic determinants to pathogens, *e.g.*, *E. coli* can transmit plasmidic AMR-genes to *Salmonella* [11], [12]. However, the *in-vivo* frequency of such transfer is unknown, and may be limited by the number of plasmids shared [12], differences in plasmid developments between bacterial species [11], or restrictions on plasmid establishment in the heterologous recipients [13]. The frequency of plasmid transfer from *E. coli* to *Salmonella* is much lower compared to promiscuous plasmid sharing between *E. coli* cells [12]. However, occasionally AMR-strains themselves exhibit a higher virulence for [14], or a greater ability to colonize animal hosts [15], [16]. This necessitates the use of even newer drugs to combat animal infections [1].

A complete cessation of antimicrobial therapies in food animals is impractical [1], and, in the absence of alternatives, unethical [17]. The real challenge is to implement therapies that minimize emergence and spread of AMR [17], [18]. Also, farm animals present a model system where the potential of candidate policies for reduction of antimicrobial usage can be evaluated at the population level, with further relevance to policies in humans [19].

The containment of resistance to 3rd generation cephalosporins is categorized by the World Health Organization as critically important. Ceftiofur is the only drug in this class licensed to treat food animals in the USA. Ceftiofur’s chemical structure is close to that of ceftriaxone, which is used to treat bacterial meningitis and salmonellosis in humans. Ceftiofur is administered parenterally to individual cattle to treat interdigital necrobacillosis, pneumonia or metritis, and to groups of beef calves for metaphylaxis of bovine respiratory disease (BRD). The drug can also be applied intramammary to treat mastitis or as a dry-off therapy.

Resistance to ceftiofur in enteric bacteria of cattle in the USA is mediated predominantly by plasmid-encoded gene *blaCMY-2* [12], [20], which codes for a cephamycinase [21], [22], [23]. The gene has been reported in *Salmonella* and *E. coli* isolates from feces of food animals and meat products in retail [24], and in *Salmonella* isolates responsible for human illness [25], [26]. The resistant *E. coli* have been isolated from feces of beef and dairy cattle, sewage and ground beef [24], [27]. Between bacteria, both inter-generational and horizontal transfers of plasmidic *blaCMY-2* occur. In the enteric environment, the horizontal plasmid transfer is the main mechanism of AMR-gene spread within and between bacterial species, both Gram-positive and Gram-negative [13]. *E. coli* can constitute up to 86% of the fecal Gram-negative bacteria in dairy cattle [28], and act as a donor of plasmidic AMR-genes [13]. Recent field studies demonstrate that a fraction of enteric *E. coli* carry plasmidic *blaCMY-*2 even in cattle not known to be treated with ceftiofur [27], [29], [30]. Enteric *E. coli* are primarily commensal and are genetically diverse [10], [31]; among them, *E. coli* carrying *blaCMY-2* are not strongly clonal at either serotype or PFGE levels [6], [12]. The “background” resistant fraction can have mixed origins. Ecological origins may include adaptation of bacteria to co-exist with fungi that are natural producers of *β*-lactams, and subsequent transfer of chromosomal AmpC locus from *Citrobacter freundii* to other *Enterobacteriaceae* as a plasmidic gene [21], [32]. Also, exposure to resistant *E. coli* can occur on the farm when post-weaned calves are colonized with ruminant-specific microflora (Tom Besser, personal communication). Similarly, ceftiofur-resistant *E. coli* in broilers is associated with its presence at the hatchery and on the farm [33].

During parenteral treatment with ceftiofur, a decline in the numbers of enteric *E. coli* is reported in healthy 3-4 mos old calves [27], healthy adult cattle [34], and lactating dairy cattle treated for metritis or interdigital necrobacillosis [35] (Table S1). Studies employing genetic methods to examine the effects on entire enteric bacterial populations have arrived at similar conclusions [36]. This strongly suggests that parenteral treatment of cattle with ceftiofur results in exposure of their enteric bacteria to antimicrobially-active drug metabolites, with the dose and duration sufficient for prominent effects on the enteric bacteria.

The objectives of this modeling study were to analyze, first, whether the reported fractions of *blaCMY-*2-carrying commensal enteric *E. coli* in cattle could be maintained in the absence of immediate ceftiofur pressure; and, second, how the dynamics of the resistant and sensitive enteric *E. coli* changed during parenteral ceftiofur treatment depending on the treatment protocol.

**Figure 1:**
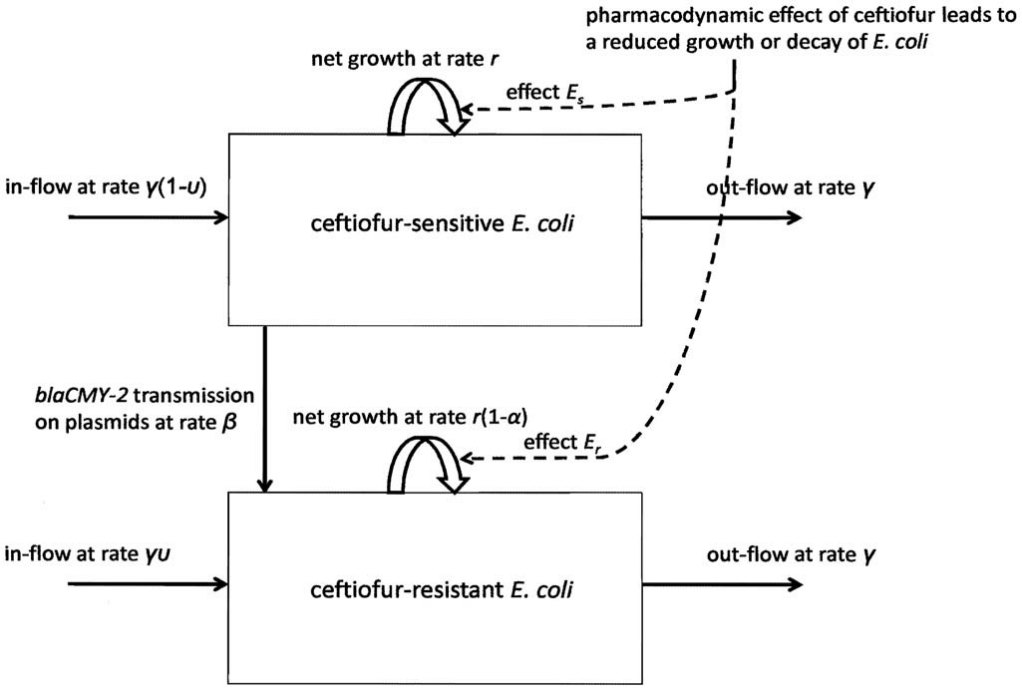
Flow-chart of the model of the dynamics of ceftiofur-sensitive and resistant commensal *E. coli* in the cattle large intestine. Bacterial growth is density-dependent with fractional net growth rate *r*; fitness cost for cells with *blaCMY-2*-carrying plasmids manifests as a reduction *α* in *r*. Resistant cells transfer *blaCMY-2* to the progeny during cell division. Horizontally, *blaCMY-2* is transferred to the sensitive cells at rate *β*; the transmission is frequency-dependent with the total of β*N_r_*N_s_/*N*. There is fractional in-flow and out-flow of *E. coli* at rate *γ*; fraction *υ* of in-flowing *E. coli* are ceftiofur-resistant. Antimicrobial action of ceftiofur metabolites, depending on their concentration, results in either reduced growth or decay in number of *E. coli*.

**Table 1:** Parameter definitions and values.

Parameter		Definition, units	Value	Reference
Bacteria				
*r*		Specific growth rate, h^−1^	0.17	estimated from [39]
*γ*		Fractional in-flow/out flow, h^−1^	0.01	estimated from [27]
*N_max_*		Max *E. coli*, log CFU/*g* of feces		
		6-mos beef (220 kg)	5.5	[31]
		6-mos dairy (180 kg)	6.5	[27]
		adult dairy (600 kg)	4.3	[90]
		post-partum/lactating dairy (600 kg)	4.3	–
AMR				
pAMR		Fraction of ceftiofur-resistant enteric *E. coli* at start of treatment		
		6-mos beef (220 kg)	0.018	[80]
		6-mos dairy (180 kg)	0.050	[78]
		adult dairy (600 kg)	0.007	[79]
		post-partum/lactating dairy (600 kg)	0.018	–
*β*		Plasmid transmission term, h^-1^	0.004	[27], [42]
*a*		Resistance fitness cost as fraction of *r*	0.05	[42]
*υ*		Resistant *E. coli* fraction in in-flow(pAMR*0.6 based on [31])		
		6-mos beef (220 kg)	0.0110	
		6-mos dairy (180 kg)	0.0310	
		adult dairy (600 kg)	0.0042	
		post-partum/lactating dairy (600 kg)	0.0110	
Biliary ceftiofur metabolites				
*p*	Bile-excreted fraction of injected dose		0.37	[47], [51]
*Tδ*	Passage time to large intestine, h		6	–
*V*	Volume of large intestine, L			
	6-mos beef or dairy		5	–
	adult cattle		20	–
*λ*	Biodegradation decay constant, h^−1^		0.2	estimated from [48], [60]
H	Hill coefficient in *E_max_* model		1.5	estimated using [68]
MICs	MIC for sensitive *E. coli*, µg/mL		1	–
MICr	MIC for resistant *E. coli*, µg/mL		8	–

## Materials and Methods

### Dynamics of Ceftiofur-sensitive and Resistant Commensal Enteric *E. coli* in the Absence of Immediate Ceftiofur Pressure

#### Ecology of commensal enteric *E. coli*

A flow-chart of the model is given in Figure 1. Due to unfavorable conditions for *E. coli* growth in the upper parts of the cattle gastrointestinal tract, only *E. coli* in the large intestine was considered (referred to as “commensal enteric *E. coli*”). These may exist in a planktonic “free-living” mode of growth, or by being incorporated into intestinal biofilms [37]. The biofilm-trapped latter likely constitute a small fraction of the total, hence enteric *E. coli* were considered to be free-living. Population growth of *E. coli*, as a facultative anaerobe, slows in anaerobic conditions [38]; the maximum net growth rate (in exponential growth phase) in numbers of enteric *E. coli*, *r*, was parameterized accordingly. *E. coli* growth in the enteric environment is likely further restricted by intra-specific competition, inter-specific competition with other microflora, and feces substrate composition [39]. A logistic model of bacterial growth was used to reflect the intra-specific competition. The upper limit for total *E. coli* per *g* of feces, *N_max_*, was parameterized from the reported numbers of viable *E. coli* in cattle feces (Table 1), and so bore the expected effects of the inter-specific competition.

*E. coli* is capable of replicating outside animal hosts; commensal *E. coli* circulate between cattle hosts and their environment [40]. In beef cattle reared at either pasture or feedlot, ∼60% of fecal *E. coli* are genetically related to those in animals’ oral cavities [31]. From *in vivo* experiments in post-weaned calves [27], an estimated ∼20–30% of fecal coliforms are *E. coli* strains fed to the animals on the day of measure or the preceding day. The in-flow of ingested bacteria and the out-flow of bacteria with feces likely ensures a regular partial replacement of *E. coli* “free-living” in the large intestine. To reflect this, the rates of hourly fractional in-flow and outflow of enteric *E. coli* were taken to be equal, both *γ*. A fraction *υ* of the ingested bacteria was assumed to carry plasmids with *blaCMY-2*. In-flowing bacteria would mix homogeneously with those already in the intestine.

#### Plasmid transfer and fitness cost of plasmid-mediated resistance

Various conjugative plasmids of *E. coli* can carry *blaCMY-*2 [12]. There is no evidence of enhanced plasmid transfer in enteric *E. coli* during parenteral ceftiofur therapy [27]. The maximum number of cells to which a donor *E. coli* can transfer a plasmid per unit time is inherently restricted by biology of conjugation; the transconjugant (recipient cell) undergoes a 40-80 minutes maturation before becoming a proficient donor [41]. Therefore, the transfer was modeled as a contagious process [65], [66] with frequency-dependent transmission. *β* was the transmission term for *blaCMY-*2-carrying plasmids from resistant donor to sensitive cells, *N_r_* - number of resistant cells, *N_s_* - number of sensitive cells, and *N* - total number of *E. coli* cells. Then, “force of transfer” per a sensitive cell per unit time was β*N_r_/*N*, and the total transfer was β*N_r_*N_s_/*N*.

The growth rate of a bacterial strain is considered to represent its evolutionary fitness [38]. Having *blaCMY-2*-carrying plasmids is associated with either a fitness cost, *i.e.*, reduced growth [42], or a fitness gain, *i.e.*, enhanced growth [43]; or no change in growth [44]. The fitness cost appears more often, and was modeled as a fractional reduction, *α*, in net growth rate, *r* [45].

The fate of AMR-bacterial strains in the absence of antimicrobial pressure is unclear. In some laboratory experiments, a gradual loss of AMR-gene-carrying plasmids during cell divisions after thousands of bacterial generations (several months) is reported [42]; others, however, report maintenance of the plasmid profile, in particular by *E. coli* [44]. The AMR-strains can acquire compensatory mutations to restore fitness without losing resistance, *e.g.*, a better growth performance of *E. coli* with chromosomal-encoded resistance to streptomycin [46], or plasmid-encoded resistance to tetracycline [43]. Notably, these processes occur over extended time horizons. The period of parenteral treatment of cattle with ceftiofur is at most 7 days, followed by at most a 13-day pre-slaughter withdrawal period. Hence the possibility of loss of plasmidic *blaCMY-2* by enteric *E. coli* was not considered in this analysis.

#### Model for dynamics without treatment

The ordinary differential Equations [1] and [2] described the changes in *N_s_* and *N_r_*, respectively, over time in the absence of immediate ceftiofur pressure:
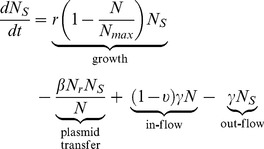
(1)

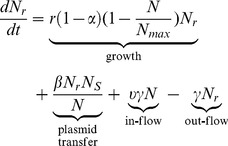
(2)


### Dynamics of Ceftiofur-sensitive and Resistant Commensal Enteric *E. coli* During Parenteral Ceftiofur Treatment

#### Pharmacokinetics and biodegradation of ceftiofur metabolites

Ceftiofur in cattle is metabolized shortly post injection (p.i.) [47]. Major ceftiofur metabolites retain the *β*-lactam ring [48]; their antimicrobial activity against *E. coli* is close to that of ceftiofur [49], [50]. The total of ceftiofur and its active metabolites is termed the concentration of ceftiofur equivalents (CE) [51]. The pharmacokinetics of ceftiofur in cattle following an intramuscular (IM) or a subcutaneous (SC) injection in cattle are similar in terms of CE-pattern in the plasma [52]. The pharmacokinetics of formulations containing ceftiofur sodium and ceftiofur hydrochloride salts are similar in terms of CE-pattern in the plasma of pigs [53]; this is considered to hold for cattle [48].

In humans, ceftriaxone (its structure is close to that of ceftiofur) is excreted via both renal and hepatic pathways [54]. There is no evidence of a correlation between ceftriaxone concentrations in bile (bile metabolite is structurally similar to ceftriaxone) and in plasma [54], or of ceftriaxone intestinal absorption and enterohepatic circulation [55]. The rate of ceftriaxone biliary excretion in humans positively correlates with the rate of bile acid secretion [56]; experimental data in rats suggest a common mechanism for hepatic transport of ceftriaxone and bile acids [57]. There is inter-individual variability in achieved ceftriaxone concentrations in bile [54], [56], [58], and in feces [54] of humans.

**Figure 2:**
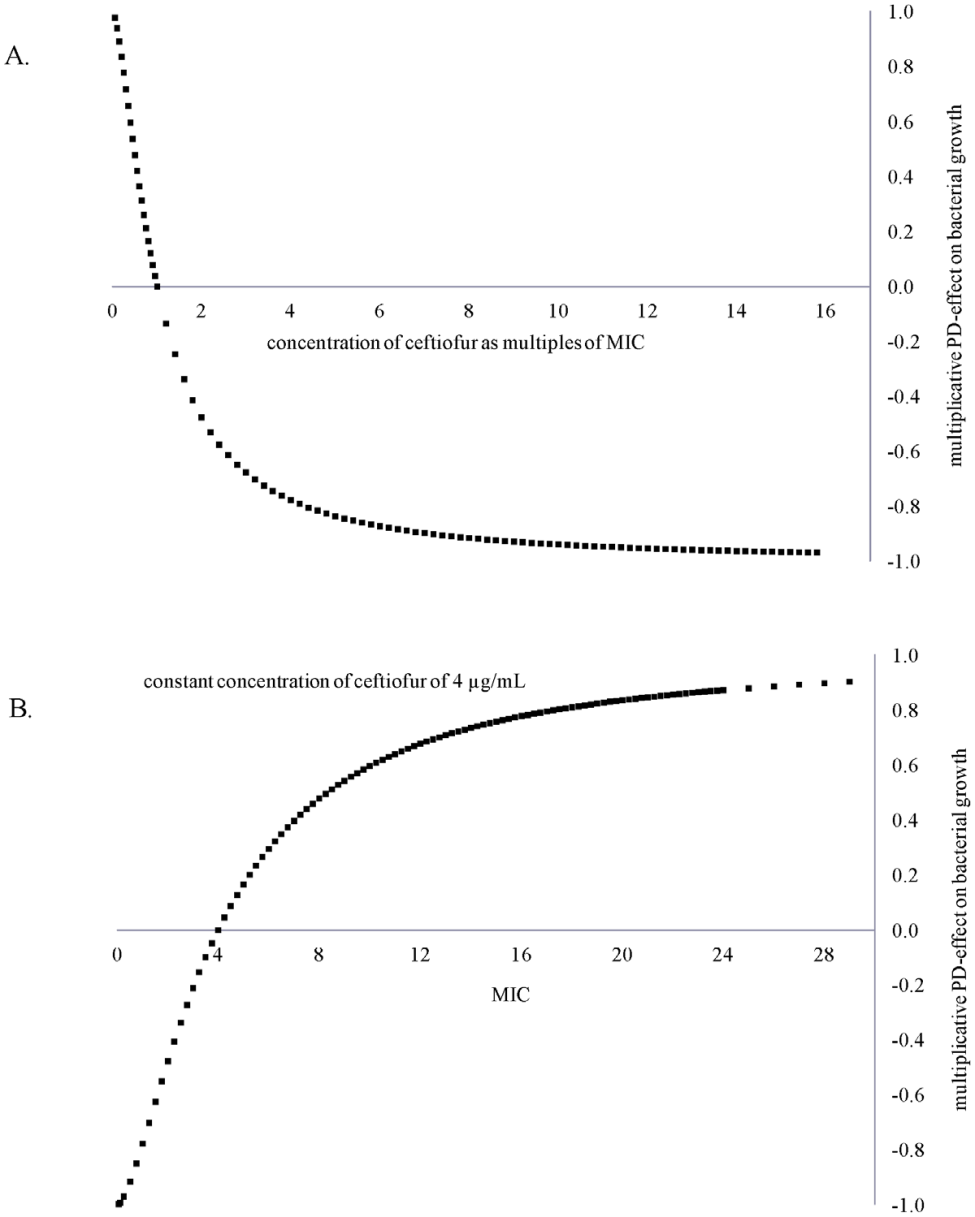
Pharmacodynamic model. A. Multiplicative pharmacodynamic effect on *E. coli* net growth with a constant minimum inhibitory concentration (MIC = 1 µg/mL) and changing ceftiofur concentration expressed as multiples of MIC; Hill coefficient = 1.5. B: Multiplicative pharmacodynamic effect on *E. coli* net growth with changing MIC and a constant ceftiofur concentration; Hill coefficient = 1.5.

In cattle, ceftiofur administered parenterally is also excreted via both urine (∼65%) and, through bile, feces (∼35%) [47]. There are no published data on the pattern or inter-individual variability of the biliary excretion. We assumed that in cattle, as in humans, there was no enterohepatic ceftiofur circulation, and CE-concentration in the intestine was independent of that in systematic distribution. Of (radio-labeled) ceftiofur dose injected IM, 29% is detected in cattle feces in 8 hours, and 37% in 12 hours p.i. [51]. The exact structure of intestinal metabolites is unknown; although it is likely that they enter the large intestine having an intact *β*-lactam ring. However, most of the (radio-labeled) amounts in feces lack antimicrobial activity [48], [59]. This is attributed to enteric bacteria biodegrading metabolites within and outside the intestinal environment [60], because of variable timing of metabolite degradation in normal *vs.* sterilized cattle feces [60]. In normal cattle feces fortified with 100 µg/*g* of CE, under aerobic conditions it takes ∼8 hours for these to entirely degrade to antimicrobially inactive compounds [48], [60]. The dynamics of decline in CE-concentration appears to be an exponential decay [48], [60]. The exact enteric species producing *β*-lactamases involved are unknown [48]; different species may be involved, as plasmid-mediated *β*-lactamases are widely produced by Gram-negative bacteria [61].

Let *D* denote ceftiofur dose in one injection. Fraction *p* of *D* was excreted in bile, and the volume of the animal’s large intestine was *V*.

First consider a therapy with repeated injections of a non-sustained-release ceftiofur formulation (Table S1, scenarios R1-R3). *D* was, and *p* and *V* were taken to be equal for every injection *n_j_*, which occurred at time *Tj* since start of treatment, *t* = 0. As the pattern of ceftiofur biliary excretion is unknown, two possibilities were explored. Under pattern 1, amount D*p was excreted at 1 hour p.i. After a passage time *Tδ*, biliary metabolites entered the large intestine. At entry, for a given *n_j_*, CE-concentration per *g* of feces (assuming weight-to-volume ratio of feces of 1) was D*p/*V*, then decayed exponentially due to the biodegradation at rate *λ*. Total CE-concentration per *g* of feces in large intestine, *C* (CE µg/g), at time *t* was:
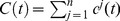
(3)

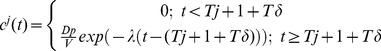
(4)where 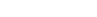; *j* = injection number: 1, …, *n*, *n* = 5

Under pattern 2, *m* = 6 equal fractions of D*p were excreted hourly at hour 1 to 6 p.i. (similarly to uniform patterns of ceftriaxone biliary excretion in rabbits [62], and of bile flow in cattle [63]). *k* was excretion fraction number. The choice of hours 1 to 6 p.i. was based on the working hypothesis that *Tδ* = 6 hours, thus the entire amount D*p would reach the large intestine by 12 hours p.i (as in experimental observations [51]). Initial CE µg/g feces was D*p/6*V. The concentration *C* (CE µg/g), at time *t* was as in Equation [3]; *c^j^* for a given *n_j_* was:
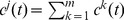
(5)

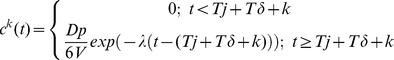
(6)where 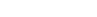, *j* = injection number: 1, …, *n*, *n* = 5 and *k* = 1,…*m*, *m* = 6.

Now consider a SC injection of a sustained-release ceftiofur formulation (Table S1, scenarios SB1-SB3 and SD1). According to the data published by the drug manufacturer, plasma CE-concentration peaks at hours 1 to 2 p.i., then declines but remains above the therapeutic threshold (0.2 µg/mL) for ∼10 days [64], [65]. Due to the quality of these data, data for 60 time points over 10 days p.i. were extracted to detail the plasma pattern. We assumed that the plasma pattern paralleled the pattern of drug release, and that entire drug dose, *D*, was released within 10 days p.i. At each time point *i*, amount *d_i_* of ceftiofur (so that 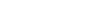; what fraction of *D* was *d_i_* was determined by the drug release pattern) was released at the site of injection. Fraction *p* of *d_i_* was excreted in bile 1 hour later. The passage of metabolites to the large intestine, and decay in their concentration due to the biodegradation were modeled similarly to the above.

**Figure 3:**
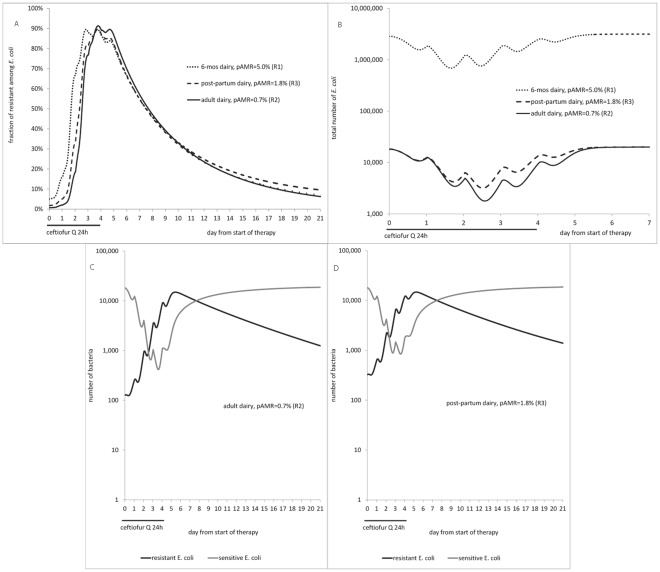
Effect of therapy with repeated ceftiofur administration on enteric *E. coli* in the deterministic case considered (*r* = 0.17, *α* = 0.05, *β* = 4^−3^, *γ* = 0.01; all h^−1^). A: Fraction of ceftiofur-resistant among *E. coli*. B: Total number of *E. coli*. C: Dynamics of ceftiofur-sensitive and resistant *E. coli* in an adult dairy. D: Dynamics of ceftiofur-sensitive and resistant *E. coli* in a post-partum dairy. pAMR = frequency of resistance at start of therapy.

**Figure 4:**
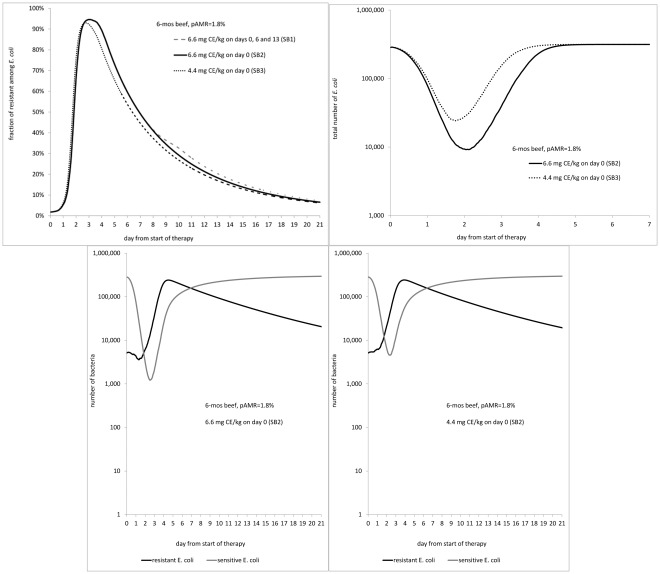
Effect of therapy using a sustained-release ceftiofur formulation on enteric *E. coli* in the deterministic case considered (*r* = 0.17, *α* = 0.05, *β* = 4^−3^, *γ* = 0.01; all h^−1^). A: Fraction of ceftiofur-resistant among *E. coli*. B: Total number of *E. coli*. Dynamics of ceftiofur-sensitive and resistant *E. coli* in a 6-mos beef treated with C: 6.6 mg CE/kg dosage, and D: 4.4 mg CE/kg dosage. pAMR = frequency of resistance at start of therapy.

#### Pharmacodynamic effect

Antimicrobial action of *β*-lactams results in the death of growing, preparing to divide bacteria (both the dividing cell and its “daughter” cell are killed); unaffected growing cells replicate (survive and produce “daughter” cells) [66]. What fraction of growing cells is killed *vs.* is replicating at a given time, and so what is net growth or decline of the bacterial population, depends on the concentration of *β*-lactams [67], [68]. The changes in net growth of ceftiofur-sensitive and resistant enteric *E. coli* depending on CE-concentration were modeled using a fractional inhibitory *E_max_* pharmacodynamic (PD) model, where *E_max_* term specifies the maximum possible PD-effect [69], [70]. The 50% PD-effect was with stationary concentration of CE, at which half of the growing cells were replicating, and half were killed (no net change in number of bacteria) [71]. In the case of *β*-lactams, for a given drug and microbe, the stationary concentration is close to a commonly measured minimum inhibitory concentration (MIC) [71]. Therefore, in this PD-model at a CE-concentration <MIC, net population growth was positive (as more growing cells were able to replicate than were killed). At a CE-concentration >MIC, the population declined. The maximum decline was when all growing cells were killed; the population declined at the rate of attempted growth. This was specified by setting *E_max_* = 2 (giving -1 as the multiplier for growth rate at a sufficiently high CE-concentration). If CE-concentration rose further, the PD-effect saturated, as no more cells could be killed than those growing to divide. The PD-model behavior is illustrated in Figure 2. The total kill depended on how long CE-concentration was at or above that producing maximum effect. The model therefore depicted time-dependent PD of cephalosporins [68], [72], [73], with a point of maximum effect (at a drug concentration of low multiples of MIC) after which further concentration rise does not enhance the rate of killing [68], [72], [74]. The model also accounted for that for antimicrobial resistance via enzymatic deactivation that can be surmounted by a higher drug dose, the change in antimicrobial activity against resistant bacteria should be reflected as an increase in the drug concentration producing the 50% PD-effect [75].

Denoting MIC_s_ for ceftiofur-sensitive and MIC_r_ for resistant *E. coli*, *E_s_* in Equation [7] and *E_r_* in Equation [8] described fractional changes in net growth of ceftiofur-sensitive and resistant *E. coli*, respectively, at CE-concentration *C*:
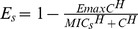
(7)

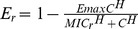
(8)where *E_max_* = 2, and *H* is Hill coefficient.

Postantibiotic effect, the period post exposure to antibiotic after which surviving bacteria begin to multiply normally, in Gram-negative bacilli after the majority of *β*-lactams is from none to brief [68], [76], [77], and so was not considered.

#### Model for dynamics during treatment

The ordinary differential Equations [9] and [10] described the changes in *N_s_* and *N_r_*, respectively, over time of parenteral ceftiofur treatment:
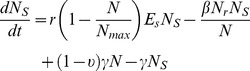
(9)

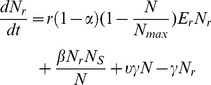
(10)


Post treatment, when ceftiofur metabolites had been eliminated from the large intestine (*C* = 0, *E_s_* = 1, *E_r_* = 1), the dynamics reverted to Equations [1] and [2].

### Parameterization

Table 1 details the parameters and their values. The maximum number of *E. coli* per *g* of feces in large intestine, *N_max_*, was based on reported numbers of viable fecal *E. coli* (colony-forming units, CFU, of *E. coli* or fecal coliforms were considered as a measurement of viable *E. coli*). Starting values of *N_s_* and *N_r_* were calculated using *N* (set at 90% of *N_max_*), and pAMR - fraction of ceftiofur-resistant *E. coli* at start of treatment. pAMR estimates were adopted from tests of *E. coli* sensitivity at start of or without connection to ceftiofur treatments. The available estimates varied depending on cattle age and purpose: 6% of ceftriaxone-resistant *E. coli* for breakpoint ≥16 µg/mL in 2-6 mos post-weaned dairy calves (estimated from [78]); 7.4% of ceftiofur-resistant *E. coli* for breakpoint ≥16 µg/mL in dairy cattle [28]; 0.7% of ceftazidime-resistant coliforms for breakpoint ≥8 µg/mL across samples from 39 dairy herds [79]; and 1.8% of ceftazidime-resistant *E. coli* for breakpoint ≥8 µg/mL in feedlot steers [80].

*E. coli* doubling time in the large intestine was assumed to be 4 hours [39]; hence hourly net growth rate in the exponential phase of population growth (in bacteriological terms, the specific growth rate), *r*, was 0.17. The fitness cost of resistance (fractional decrease in *r*) was parameterized from *in vitro* competition assays between *E. coli* strains carrying plasmids with *blaCMY-2* and those that do not; a crude average of experimental data *α* = 0.05 was used [42].

Rates of horizontal transfer of individual plasmids with *blaCMY-*2 *in vitro* vary from 10^−8^ to 10^−3^ [42], [81]. *In vivo* in post-weaned calves fed a donor and a recipient *E. coli* strains, the overall rate of generation of *blaCMY-2-*transconjugates in fecal *E. coli* is 8^−5^ to 2^−3^ [27].

The rates of hourly fractional in-flow and out-flow of *E. coli* “free-living” in the large intestine, *γ* = 0.01 (to the daily total of 0.24), were estimated from an *in vivo* study in post-weaned dairy calves [27]. The fraction of ceftiofur-resistant cells in in-flow was set at 0.6*pAMR, based on 60% genetic similarity of *E. coli* in oral cavities and in feces of beef cattle reared at either pasture or feedlot [31].

Of parenteral ceftiofur dose, *D*, fraction *p* = 0.37 was excreted in bile within 6 hours p.i. (under excretion pattern 1 or 2) [51]; metabolites reached the large intestine in *Tδ* = 6 hours post excretion. Volume of the large intestine was 20L in an adult cattle, and 5L in a 6-mos calf. The rate of exponential decay in CE-concentration in the large intestine was twice lower compared to feces under aerobic conditions [48], [60].

As there are no published data from a time-kill experiment for *E. coli* and ceftiofur, the PD-model was applied to reproduce the data from *in vitro* time-kill experiments for *E. coli* and *β*-lactam ticarcillin [68], and performed well; *H* of 1.5 performed optimally for both concentrations below and above MIC. Under aerobic conditions, ceftiofur and its major metabolites are highly active against veterinary isolates of *E. coli*, with MIC_50_ = 0.25 µg/mL and MIC_90_ = 0.50 µg/mL [50]. Decrease in activity under anaerobic conditions appears to be limited (“one 2-fold dilution” *in vitro*), but the data are scarce. For the PD-model, MIC_s_ = 1 µg/mL and MIC_r_ = 8 µg/mL were used.

**Figure 5:**
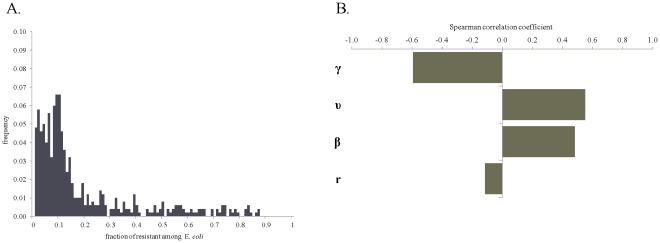
Fraction of ceftiofur-resistant among enteric *E. coli* in long-term absence of ceftiofur pressure. A: Uncertainty: frequency histogram for 500 model simulations with randomly varying parameters of bacterial ecology. B: Sensitivity: significant linear correlations (*p*-value ≤0.05) between ranked-transformed values of the parameters of bacterial ecology and the fraction of resistance.

**Figure 6:**
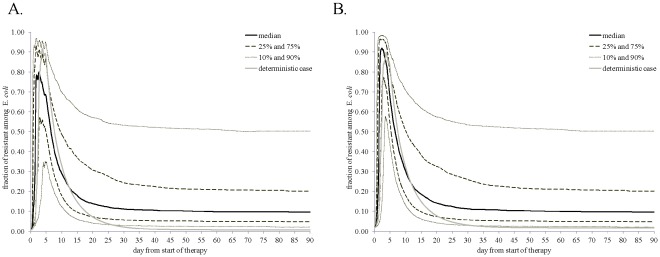
Uncertainty in the fraction of ceftiofur-resistant among enteric *E. coli* during 90 days from start of therapy. Statistics are for 500 model simulations at each time point randomly varying parameters of bacterial ecology and pharmacodynamics. A: A 5-day repeated ceftiofur administration to an adult dairy (frequency of resistance at start of therapy 0.7%). B: An injection of a sustained-release ceftiofur formulation to a 6-mos beef (frequency of resistance at start of therapy 1.8%).

Sensitivity of methods based on bacteriological culture to detect a strain of *E. coli* in bovine feces is generally restricted to when >100 CFU/*g* is present [82]. We processed model outputs, *N_s_* and *N_r_*, to separate scenarios when ceftiofur-resistant *E. coli* likely would not be detected by culturing the feces.

### Model Solving, and Uncertainty and Global Parameter Sensitivity Analysis

Solutions of the ordinary differential equations were approximated numerically using the fourth-order Runge–Kutta method implemented in Vensim® PLE Plus software (Ventana Systems, Inc.; Harvard, MA, USA). In the deterministic analysis, first, for each treatment scenario (Table S1), the model without treatment (Equations [1] and [2]) was solved varying parameter values to reproduce the reported pAMR (Table 1). Then concentration *C*, and PD-effects *E_s_* and *E_r_* were calculated. These were introduced into the model (Equations [9] and [10]) and the equations were solved. The models were solved starting with total *E. coli*, *N*, at 90% of *N_max_*.

The analysis of uncertainty and global parameter sensitivity of the model outputs was conducted for the treatment scenarios R2 and SB2 (Table S1). Given the dynamics of ceftiofur metabolites in the large intestine during therapy (section *Pharmacokinetics and biodegradation of ceftiofur metabolites* above), the sensitivity analysis was targeted at how the model outputs correlated with changes in the parameters of ecology of enteric *E. coli* (*r*, *α, β*, *γ* and *υ*), and of pharmacodynamics of the metabolites against *E. coli* (MIC_s_, MIC_r_ and *H*). A uniform distribution (*U*) was assumed for all, because of the lack of knowledge of distributions of individual parameters. The minimum and maximum values were specified based on the literature review (all rates h^−1^): *r*∼*U*(0.05, 0.5), *α*∼*U*(−0.2, 0.2), *β*∼*U*(10^−5^, 0.01), *γ*∼*U*(10^−3^, 0.02), *υ*∼*U*(10^−5^, 0.10), MIC_s_∼*U*(0.2, 1.9), MIC_r_∼*U*(2, 16), and *H*∼*U*(0.5, 4). For each of the two treatment scenarios, 500 Monte Carlo simulations were performed with Latin Hypercube sampling of each parameter space at each time point over 90 days from start of therapy for the model with treatment, and for 180 days for the model without treatment. This was implemented in Vensim® PLE Plus software. The uncertainty in model outputs was explored graphically. The sensitivity of model outputs to changes in values of each parameter was evaluated with the Spearman correlation coefficient (*ρ*) [83]. Whether *ρ* was significantly different from zero was tested with a Student’s *t*-test, with the test statistics, denoting the number of simulations as *w,* calculated as 
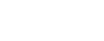
 and assumed to follow a *t*-distribution with (*w*-2) degrees of freedom. If the test’s *p*-value was ≤0.05, the correlation was considered as significant.

## Results

### Deterministic Analysis

#### Maintenance of ceftiofur resistance in commensal enteric *E. coli* in the absence of immediate ceftiofur pressure

In the model without treatment (Equations [1]–[2]), for every scenario (Table S1) the reported pAMR could be reproduced with *r* = 0.17, *α* = 0.05, *β* = 4^−3^, *γ* = 0.01, and *υ* = 0.6*pAMR. (These parameter values were used for the deterministic analysis of treatment scenarios: Table 1). *N_max_* corresponded to the scenario, and had no influence on the dynamics observed. pAMR was most sensitive to *β* and *υ* (or, if *υ* was kept constant, to *γ*). *β*>0.01 (somewhat unrealistic, see the discussion below) allowed reproducing the reported pAMR if there was no ceftiofur resistance among ingested *E. coli*, *υ* = 0. In all the scenarios, the resulting *N_r_* was over 100; hence, culture-based methods would likely detect the presence of resistance.

#### Effects of parenteral ceftiofur therapy on commensal enteric *E. coli*: repeated ceftiofur administration

Models with both hypothesized biliary excretion patterns produced outputs resembling experimental data (Table S1); thus, biliary excretion of ceftiofur likely occurs within the first several hours p.i. Further results and discussion refer to the model with excretion pattern 2 (uniform excretion hourly at hour 1 to 6 p.i.).

**Figure 7:**
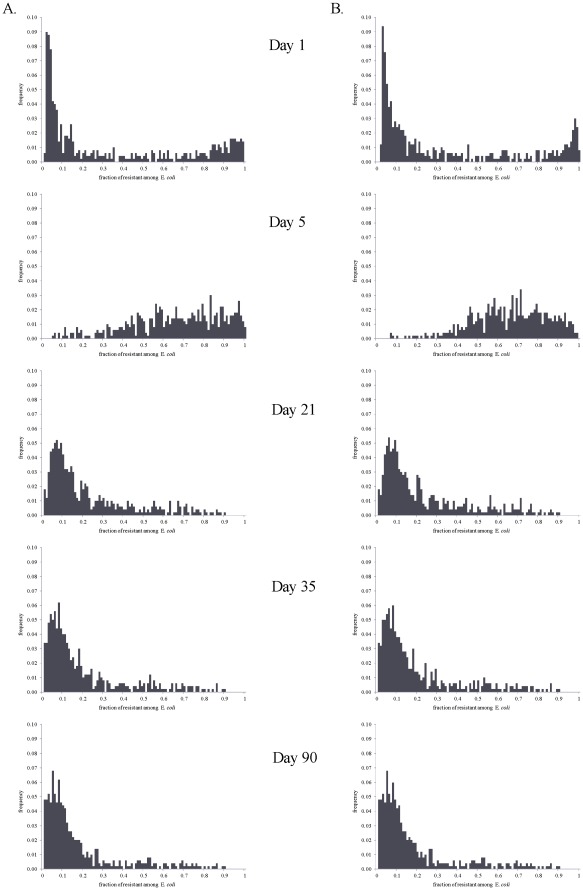
Frequency histograms for the fraction of ceftiofur-resistant among enteric *E. coli* on days 1, 5, 21, 35 and 90 from start of therapy. Histograms are of 500 model simulations at each time point randomly varying parameters of bacterial ecology and pharmacodynamics. A: A 5-day repeated ceftiofur administration to an adult dairy (frequency of resistance at start of therapy 0.7%). B: An injection of a sustained-release ceftiofur formulation to a 6-mos beef (frequency of resistance at start of therapy 1.8%).

**Figure 8:**
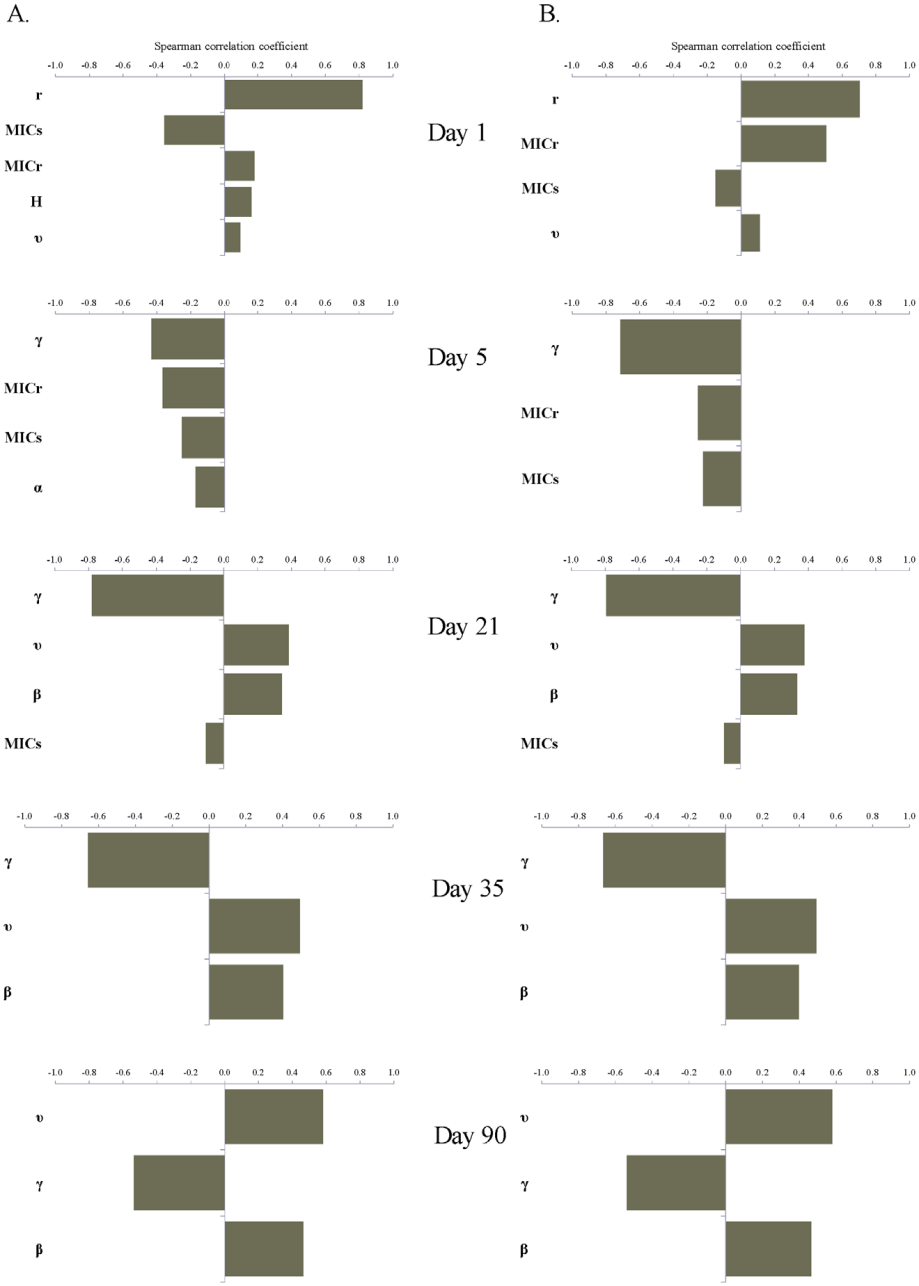
Significant linear correlations (*p*-value ≤0.05) between ranked-transformed values of the parameters of bacterial ecology and pharmacodynamics and the frequency of ceftiofur-resistant among enteric *E. coli* on days 1, 5, 21, 35 and 90 from start of therapy. A: A 5-day repeated ceftiofur administration to an adult dairy (frequency of resistance at start of therapy 0.7%). B: An injection of a sustained-release ceftiofur formulation to a 6-mos beef (frequency of resistance at start of therapy 1.8%).

For all the treatment scenarios, the model (Equations [9]–[10]) outputs showed a decrease in counts of total and of ceftiofur-sensitive enteric *E. coli*, and a rise in the fraction of resistant among *E. coli* during therapy, similar to experimental observations (Fig. 3 *cf.* Table S1, scenarios R1-R3). For a 5-day therapy in an adult dairy, the model showed a decrease in total count from day 1, to a minimum on day 3, remaining decreased until day 4, and then returning to pre-treatment level on day 7 (Fig. 3B). The timing was close to the results for an on-farm 5-day ceftiofur therapy reporting fecal *E. coli* decreasing from day 1, to a minimum on day 6 (the samples were not collected every day), returning to pre-treatment level by day 9 (Table S1, R2). The maximum drop differed between the model, ∼1.05 log CFU/g, and the on-farm study, estimated ∼4.0 log CFU/g. However, the count change in the model corresponded to a 91% reduction in number of *E. coli*. Also, there may have been inter-individual variability in the count and its dynamics in the cattle treated on-farm, but only average numbers were reported. For a 5-day therapy in a 6-mos dairy, the model output of a 0.66 log CFU/g drop in total *E. coli* resembled well the field data of 0.5-1 log CFU/g drop in culturable fecal bacteria in individual 2-6 mos calves [78] (Fig. 3B *cf.* Table S1, R1). In both these scenarios, the total *E. coli* count dropped starting from day 1 of therapy.

As total *E. coli* was composed of both ceftiofur-sensitive and resistant cells, propagation of the latter offset the decline in total count, but this took time. *E.g,* in an adult dairy the total *E. coli* count was lowest on day 3 of therapy, dropping by 1.05 log CFU/g (Fig. 3B); but the count of ceftiofur-sensitive cells was lowest on day 4, dropping by 1.68 log CFU/g (Fig. 3C). These corresponded to a 91% and 98% reduction in number of bacteria, respectively. The fraction of resistant among *E. coli* peaked on day 4 (Fig. 3A). At this point, resistant cells filled most of the “carrying capacity”, sensitive cells grew less and were less exposed to antimicrobial action (hence no further reduction in sensitive counts). Similarly, in a 6-mos calf the number of sensitive *E. coli* dropped to its minimum and the fraction of resistant *E. coli* rose to its maximum (Fig. 3A) the next day after the maximum drop in total *E. coli* (Fig. 3B).

In cattle of a given age treated under a given protocol, the lower was the initial frequency of resistance, pAMR, the larger was the decline in total count of *E. coli* (Fig. 3B, R2 *vs.* R3) and of sensitive *E. coli* (Fig. 3C *vs*. 3D) during therapy. However, the maximum fraction of resistant among *E. coli* during therapy did not seem to depend on pAMR in the range explored, peaking to 90-91% in either an adult (pAMR = 0.7%), post-partum (pAMR = 1.8%), or 6-mos (pAMR = 5.0%) dairy (Fig. 3A). Yet it took longer for the fraction to completely return to pre-treatment level if this already had been elevated, *e.g.*, ∼16 weeks in a 6-mos (pAMR = 5.0%) *cf.* ∼10 weeks in an adult (pAMR = 0.7%) dairy.

Scenarios R1-R3 were repeated with ceftiofur dosage 1.1 mg CE/kg (with biliary excretion pattern 1). In an adult dairy, including post-partum, there was a slightly lesser peak in number and relative fraction of resistant *E. coli*, and a day later in therapy compared to using 2.2 mg CE/kg. There was however a difference in a 6-mos dairy; the drop in total *E. coli* was 26% with 1.1 mg *vs.* 64% with 2.2 mg dosage, and the fraction of resistant cells rose to 27% *vs.* 80%, returning to pre-treatment level in 104 *vs.* 110 days, respectively.

#### Effects of parenteral ceftiofur therapy on commensal enteric *E. coli*: sustained-release ceftiofur formulation

For a 6-mos beef administered a sustained-release ceftiofur formulation, lowering the dosage to 4.4 from 6.6 mg CE/kg (Table S1, SB3 *vs.* SB2), resulted in a smaller drop in total *E. coli*, 1.11 *vs.*1.54 log CFU/g, occurring a day earlier, day 2 *vs.* day 3, of therapy (Fig. 4B). The count of sensitive *E. coli* dropped by 1.83 log CFU/g with 4.4 mg *vs.* by 2.4 log CFU/g with 6.6 mg (98.5% *vs.* 99.6% reduction in number of sensitive bacteria), in either case being the lowest on day 3 (Fig. 4D *vs.* 4C). Quantitatively, the decrease in total *E. coli* matched field data; the timing was not contrasted because in the field study available for comparison with the scenarios SB1-SB3, the fecal samples were only obtained on days 0, 2, 6, 9, 13, 16, 20, and 28 of therapy [84]. The fraction of resistant *E. coli*, however, still rose to its highest of 93% on day 3 with 4.4 mg, *cf.* 95% on day 4 with 6.6 mg (Fig. 4A). The fraction reported by the field study varied from 40% to 90% [84]. In both scenarios SB2 and SB3, it took 111-112 days for the fraction of resistant *E. coli* to completely return to pre-treatment level.

With 6.6 mg CE/kg dosage, whether this was administered once on day 0, or 3 times on days 0, 6 and 13 (Table S1, SB1 *vs.* SB2) had a very limited effect on the dynamics observed (Fig. 4A). This is because the fraction of resistant *E. coli* was still high, and the number of sensitive *E. coli* depressed, following the 1st dose at the time the 2nd dose was given; similarly for doses 2 and 3. This agreed with field observations [84].

For an adult dairy (SD1), the model outputs were similar to a single administration of sustained-release ceftiofur formulation in 4.4 mg CE/kg dosage to a 6-mos beef (SB3).

#### Sensitivity of model outputs to variability in parameter values

In the absence of immediate ceftiofur pressure, the maintenance of a fraction of ceftiofur-resistant cells among commensal enteric *E. coli* depended on the rate of in-flow and out-flow of *E. coli*, the prevalence of ceftiofur resistance in the in-flow, and the rate of transfer of *blaCMY-2*-carrying plasmids between *E. coli* within the intestine, and, to a lesser extent, on the rate of bacterial growth (Fig. 5B). Certain, although infrequent, combinations of the parameters produced a largely elevated frequency of resistance (Fig. 5A).

There were similar tendencies in the dynamics of ceftiofur-resistant enteric *E. coli* under the scenarios of a 5-day treatment of an adult dairy with a non-sustained release ceftiofur formulation in 2.2 mg CE/kg dosage (R2), and a single injection of a sustained-release ceftiofur formulation to a 6-mos beef in 6.6 mg CE/kg dosage (SB2) (Fig. 6). However, the median (over 500 model simulations) peak fraction of resistant among enteric *E. coli* was in the upper 70% range in the former *vs.* over 90% in the latter scenario (Fig. 6A *vs.*6B). There was a substantial uncertainty in the fraction of resistant *E. coli*, as during so after treatment, with the explored parameter ranges (Figs. 6 and 7). The largest uncertainty was observed on days 1 and 5 from start of therapy (Fig. 7). At those time points the variability in both the ecology of enteric *E. coli*, and in antimicrobial action of enteric ceftiofur metabolites against *E. coli* contributed to the outcome. On day 1 (Fig. 8), a higher fraction of resistance strongly correlated with a higher rate of bacterial growth, and a lower MIC_s_, which would correspond to a larger kill of growing sensitive *E. coli*, hence a larger niche for expansion of the resistant cells, in turn ensured by the higher growth potential. A higher fraction of resistance also correlated with a higher MIC_r_, *i.e.*, a larger expansion of resistant *E. coli* if these were less sensitive at the start of therapy. The importance of individual parameters changed by day 5 when the fraction of resistance, after reaching its maximum, was still correlated with the pharmacodynamical parameters, but also became influenced again by the rate of fractional replacement of enteric *E. coli* (Fig. 8). By day 35, the fraction of resistance tended to settle at lower than 20%, further clustering toward lower values by day 90 (Fig. 7). This outcome depended on the same parameters that were most important for resistance maintenance in the absence of treatment: the rate of horizontal transfer of *blaCMY-2*, the rate of in-flow and out-flow of enteric *E. coli*, and the prevalence of ceftiofur-resistance in the in-flow (Fig. 8, Days 35 and 90 *vs.* Fig. 5B).

## Discussion

This modeling study suggested that ceftiofur-resistant commensal enteric *E. coli* in cattle could persist between treatments. A low but stable fraction of resistance can be maintained even if the number of resistant *E. coli* grows slower than that of the sensitive ones, when the rate of *blaCMY-2* transfer in enteric *E. coli* is sufficiently high or a sufficient fraction of ingested *E. coli* is ceftiofur-resistant. The latter could occur if the conditions on the farm allow for a close circulation of commensal *E. coli* between cattle and their environment.

The values reported by field studies of the fraction of ceftiofur-resistant cells among fecal *E. coli* in the absence of immediate ceftiofur pressure were reproduced in the deterministic analysis with a transfer rate for *blaCMY-2-*carrying plasmids of 4^−3^. For individual plasmids, *in vitro* transfer rates are up to 2^−3^ [42]. *In vivo* in post-weaned calves fed a donor and a recipient strain, a rate of *blaCMY-2*-transconjugant generation in fecal *E. coli* of 2^−3^ has been reported [27]. Several plasmids may be present in enteric *E. coli*, and the *blaCMY-2*-transfer rate *in vivo* may be the cumulative of those for individual plasmids. Generally, conjugation frequency may depend on the physiological status of donor cells, phase of growth of donor population [85], and physical conditions [13], [86], [87]. As the enteric environment is *E. coli*’s ecological niche, the cells are likely in normal physiological condition; this, coupling with nutrient availability for population growth, absence of light and favorable temperatures, may allow for the bacterial conjugation, and so plasmid transfer rate, to be at the high end of the biological maximum [87], [88].

Ceftiofur-resistant *E. coli* in this study were defined as cells having a plasmid with *blaCMY-2*. A possibility of variability in the degree of resistance conferred by presence of more than one copy of the gene, or by presence of another mechanism of resistance, and how this may be reflected in MIC-values, was not considered; neither are such data available in experimental literature. This complicated interpretation of the correlations of MIC_r_ with the fraction of resistant among *E. coli* during therapy (Fig. 8).

The increase in absolute number of *E. coli* cells carrying *blaCMY-2* per *g* of feces during parenteral ceftiofur therapy could lead to a higher frequency of horizontal transfer of this plasmidic gene to the other enteric bacteria, including potential zoonotic pathogens. However, this would require not only the donor but also the recipient populations to be present in sufficient numbers [89]. The numbers of ceftiofur-sensitive cells among the other bacteria may be diminishing during therapy, similar to the numbers of ceftiofur-sensitive *E. coli*. Hence, the net effect on the frequency of *blaCMY-2*-transmission from *E. coli* to the other bacteria would depend on the degree of sensitivity of the latter to antimicrobial action of enteric ceftiofur metabolites. Importantly, the only origin and spread of AMR considered here for ceftiofur and *E. coli* were the *a-priori* presence of plasmidic *blaCMY-2*, and its vertical and horizontal transfers, respectively. Other mechanisms, *e.g.* resistance mediated by chromosomal genes or that due to plasmid-mediated extended-spectrum beta-lactamases, may need to be considered to understand AMR dynamics across the species. Therefore, based on the current model, we could not infer what effect ceftiofur therapy might have on inter-species spread of *blaCMY-2.* Furthermore, evaluating the potential for spread of AMR-determinants requires accounting for not only the within-host dynamics addressed here, but also how the resistant bacteria spread among the hosts, and between the hosts and their environment.

This study highlighted that results of ceftiofur treatment trials would be more informative for modeling if the data reported would include the dynamical change in fraction of the resistant fecal bacteria, and description of variability among individual cattle. Frequent sampling during treatment would help with detailing the length of time available for expansion of resistant bacteria; continuing sampling post treatment would help with understanding the mechanisms involved in resistance maintenance. On the epidemiological side, important knowledge gaps are details of *E. coli* cycling between cattle and their environment, including the degree of replacement of enteric *E. coli*, the prevalence of ceftiofur resistance in *E. coli* ingested by cattle, and the rates of horizontal *blaCMY*-2-transfer *in vivo.* Absence of publicly accessible data on the concentration and antimicrobial activity of ceftiofur metabolites in cattle intestine during parenteral therapy hinders more detailed research into the selective pressure experienced by enteric bacteria. On the pharmacological side, time-kill experiments (as opposed to experiments establishing MIC-values) mimicking intestinal conditions are needed to describe the pharmacodynamics of ceftiofur metabolites against enteric bacteria.

To conclude, first, the results showed that reported low fractions of ceftiofur-resistant commensal enteric *E. coli* in cattle could be maintained without immediate ceftiofur pressure. Second, during parenteral ceftiofur therapy there likely are antibiotically-active drug metabolites in the large intestine, circumventing a slash in the number of ceftiofur-sensitive enteric *E. coli*. These conclusions are strongly supported by the concordance of the model outputs with experimental data. Hence, there is a window during therapy when ceftiofur-resistant *E. coli* could expand in absolute number and relative frequency; the degree of expansion depends on the parameters of antimicrobial action of the metabolites against *E. coli*, as well as on the rates of enteric *E. coli* growth and replacement. However, whether the post-treatment fraction of resistance would remain elevated in the long-term depends on a present combination of the parameters of bacterial ecology, the same parameters that are important for maintenance of resistance in the absence of ceftiofur pressure. Namely, these are the rate of horizontal transfer of plasmids with *blaCMY*-2 between enteric *E. coli*, which may be determined by which plasmids are present, and the frequency of resistance in *E. coli* ingested by cattle, which may be determined by the extent of *E. coli* circulation between cattle and their environment.

## Supporting Information

Table S1 Modeled scenarios of treatment of cattle with ceftiofur, and experimental data considered for comparison.
(DOCM)
