# Supplementary material for: Mathematical Model of Plasmid-Mediated Resistance to Ceftiofur in Commensal Enteric Escherichia coli of Cattle
Source: PLoS One. 2012 May 16;7(5):e36738. doi: 10.1371/journal.pone.0036738 (PMC3353932; doi:10.1371/journal.pone.0036738)
Supplement: Table S1 — Modeled scenarios of treatment of cattle with ceftiofur, and experimental data considered for comparison. (DOCM) [file pone.0036738.s001.doc]

**Supporting information Table 1.** Modeled scenarios of treatment of cattle with ceftiofur, and experimental data considered for comparison.

| Scenario |  | | Experimental data | |
| --- | --- | --- | --- | --- |
|  | Type of animal, age, bw^a^; treatment | Dose/kg bw, administration (pre-slaughter withdrawal period) | Study description | Results (day 0 – start of treatment) |
| SB1 | Beef 6 mos, 220 kg; metaphylaxis of BRD^b^ | 6.6 mg CE/kg, SC in mid 1/3 of ear; 3 times: on days 0, 6 and 13 (13 days) | Beef steers treated as SB1, SB2, SB3; fecal samples on some of days 0 to 28; fecal *E. coli* enumerated, sensitivity to ceftiofur tested [[84](#_ENREF_84)] | ↓ *E. coli* counts on days 2 to 9, max by ~1.1 log CFU/g on days 2 and 16. ↑ resistant *E. coli* counts on days 6 to 20, max by ~1.4 log CFU/g on days 9 and 20. Max proportion of resistant *E. coli* (breakpoint ≥8 µg /mL) ~90% on day 20. |
| SB2 | - | 6.6 mg CE/kg, SC in mid 1/3 of ear; once (13 days) |  | ↓ fecal *E. coli* counts on days 2 to 6, max by ~ 1.2 log CFU/g on day 2. ↑ resistant *E. coli* counts on days 6 to 9, max by ~0.8 log CFU/g on day 6. Max proportion of resistant *E. coli* (≥8 µg /mL) ~40% on day 6. |
| SB3 | - | 4.4 mg CE/kg, SC in mid 1/3 of ear; once (13 days) |  | ↓ *E. coli* counts on days 2 to 9, max by ~ 1.0 log CFU/g on day 9. ↑ resistant *E. coli* counts on days 6 to 9, max by ~1.1 log CFU/g on day 6. Max proportion of resistant *E. coli* (≥8 µg /mL) ~70% on day 9. |
| SD1 | Dairy lactating, 600 kg; treat pneumonia | 6.6 mg CE/kg, SC in base of ear; once (13 days) | Non available | Non available |
| R1 | Dairy 6 mos, 180 kg; treat interdigital necrobacillosis, BRD | /2.2 mg CE/kg, IM, Q 24 h, 5 days (3 days) | 3-4 mos dairy calves; treated for 5 days, 2.2 mg CE/kg, Q 24 h; fecal samples on some of days -7 to 14; fecal coliforms and *E. coli* enumerated, sensitivity to ceftazidime tested [[27](#_ENREF_27)] | ↓ coliform counts from day 1 to min on day 2 (by ~1.2 CFU/g), returned to pre-treatment level by day 9. Correspondingly, proportion of resistant *E. coli* transiently ↑. |
|  |  |  | 2-6 mos dairy calves; treated for 5 days, 2.2 mg CE/kg, Q 24 h; fecal samples on some of days -1 to 17; fecal bacteria enumerated, sensitivity to ceftriaxone tested [[78](#_ENREF_78)] | ↓ fecal bacteria counts on day 1 by 0.5-1 log CFU/g, remained so until day 17, except for 1 log CFU/g spike on day 10. ↑ resistant bacteria counts (by 1.05, 0.87, 0.58 log CFU/g, for breakpoint ≥16, 64, 128 µg /mL respectively), remained elevated until day 17. |
| R2 | Dairy adult, 600 kg; treat interdigital necrobacillosis, BRD | 2.2 mg CE/kg, IM, Q 24 h, 5 days (3 days) | Dairy cows treated for infertility due to *Leptospira* for 5 days, 2.2 mg CE/kg, Q 24 h; fecal samples on some of days -1 to 32; fecal *E. coli* enumerated, tested for sensitivity to ceftiofur and other, overall antibiotic resistance index (ARI) calculated [[34](#_ENREF_34)] | ↓ *E. coli* counts from day 1, to min on day 6 (by ~4.0 CFU/g), returned to pre-treatment level by day 9. Overall resistance, ARI, of *E. coli* ↑ from day 3, to max on day 5, returned to pre-treatment level by day 9. |
| R3 | Dairy postpartum, 600 kg; treat acute postpartum metritis | 2.2 mg CE/kg, IM, Q 24 h, 5 days (3 days) | Cows (95% within 2 wks after calving) treated for 4 days, 1g CE, Q 24 h; fecal samples on some of days 0 to 28; fecal *E. coli* enumerated, sensitivity to ceftiofur tested [[35](#_ENREF_35)] | ↓ *E. coli* counts on days 2 and 7. Proportion of ceftiofur-resistant *E. coli* ↑ on day 2 in some cattle (from 0-2% to 6-14% for breakpoint ≥16 µg/mL). |

^a^ bw=body weight; CE = ceftiofur equivalents; IM = intra-muscular injection; SC = subcutaneous injection; Q 24 h = every 24 hours.

^b^ BRD=bovine respiratory disease
